# COVID-19 mortality dynamics: The future modelled as a (mixture of) past(s)

**DOI:** 10.1371/journal.pone.0238410

**Published:** 2020-09-11

**Authors:** Samuel Soubeyrand, Mélina Ribaud, Virgile Baudrot, Denis Allard, Denys Pommeret, Lionel Roques

**Affiliations:** 1 INRAE, BioSP, Avignon, France; 2 Univ Lyon, UCBL, ISFA LSAF EA2429, Lyon, France; University of Illinois College of Veterinary Medicine, UNITED STATES

## Abstract

Discrepancies in population structures, decision making, health systems and numerous other factors result in various COVID-19-mortality dynamics at country scale, and make the forecast of deaths in a country under focus challenging. However, mortality dynamics of countries that are ahead of time implicitly include these factors and can be used as real-life competing predicting models. We precisely propose such a data-driven approach implemented in a publicly available web app timely providing mortality curves comparisons and real-time short-term forecasts for about 100 countries. Here, the approach is applied to compare the mortality trajectories of second-line and front-line European countries facing the COVID-19 epidemic wave. Using data up to mid-April, we show that the second-line countries generally followed relatively mild mortality curves rather than fast and severe ones. Thus, the continuation, after mid-April, of the COVID-19 wave across Europe was likely to be mitigated and not as strong as it was in most of the front-line countries first impacted by the wave (this prediction is corroborated by posterior data).

## Introduction

COVID-19 currently generates a major pandemic that has caused about 350,000 registered deaths by May 28, 2020. The first cases were reported in Hubei province (China) and the epidemic has then spread around the world, with variable dates of emergence (e.g., ranging from mid-January to late February in European countries [1]). The idea of using past trends in foreign countries to predict the future trend in a given focal country has already been mentioned [2] and is classically used by media to help citizens perceive the future of an outbreak. If this idea faces up the large diversity and the numerous particularities of epidemic dynamics at country level [3], it deserves to be implemented in a formal statistical framework since mortality dynamics abroad can be viewed as real-life predictors naturally accounting for crucial factors such as population structures, health systems and control measures.

Using observations in countries that are *ahead of time* with respect to the country under focus (i.e., countries with larger current numbers of deaths, relatively to population sizes), we propose a statistical framework grounded on a flexible mixture model (i) for short-term, real-time forecasting mortality from COVID-19, but also (ii) for comparing mortality trajectories between countries. The latter point allows drawing medium-term predictions: because of the inertia of COVID-19-like epidemics that we observe (and possibly relatively similar decision making), countries that follow the past dynamics of other countries are condemned to repeat them, at least to some extent. The proposed framework and the accompanying web app http://covid19-forecast.biosp.org can help in assessing the future intensity of the COVID-19 wave as it goes on through the world and could help, in the postpandemic horizon, in screening the impact of decision making on mortality with country-to-countries comparisons. Our approach can also be implemented at a finer geographical resolution, at least for countries with large enough numbers of deaths allowing the observation of significantly different temporal signals in different regions.

The online web app provides forecats and comparisons for about 100 countries, states and provinces. It is particularly useful in the current period (May–June 2020) to screen the COVID-19 outbreaks in South American countries while South America has been declared as the new epicenter of the disease by the World Health Organization (on May 22). Thereafter, we apply our approach to compare the mortality trajectories of European countries in the front and second lines of the COVID-19 wave. We carry out this analysis with data collected up to April 20, while the wave was particularly strong in the European region. At that time, some countries in Europe were already largely impacted in terms of mortality rate (i.e., the front-line countries), while the remaining countries were less affected (i.e., the second-line countries). In addition, we assess the short-term forecast performance of the mixture model briefly introduced above with COVID-19 data (the forecast made at a given time being compared to actual data after this time). These data allow us to compute the performance of our approach in diverse real situations instead of synthetic simulated situations. Moreover, the mixture model is compared to a SIRD compartmental model including susceptible, infectious, recovered and dead individuals using two distributional assumptions for the observations (Poisson and negative-binomial) and to a log-linear regression model. The former model exploits only data from the country of interest, whereas the latter model exploits data from the country of interest as well as data from abroad, like our mixture model.

## Material and methods

### Worldwide COVID-19 mortality data

Mortality data are obtained from the Johns Hopkins University CSSE [3] and The Covid Tracking Project (https://covidtracking.com), which provide, in particular, daily mortality data at the level of countries, states or provinces, grounded on diverse data sources including the World Health Organization and national government health departments. Here, we use data from the 15 European countries having at least 200 registered deaths by April 12, 2020 (namely, Austria, Belgium, Denmark, France, Germany, Ireland, Italy, Netherlands, Poland, Portugal, Romania, Spain, Sweden, Switzerland, United Kingdom), and from Hubei province in China. Hubei was the first COVID-19 hotspot.

### Selecting countries used as predictors

Given a focal country labelled 0, whose mortality dynamics have to be forecast, the countries *i* with a larger death rate up to the *current date τ* are considered as being ahead of time, and can be chosen as predictors. Denoting by *Y*_i_(*t*) the cumulative number of deaths at date *t*, and *s*_*i*_ the population size in country *i*, this condition corresponds to *Y*_*i*_(*τ*)*s*_*i*_>*Y*_0_(*τ*)/*s*_0_ and can be easily checked in practice directly from raw data. The number *n* of possible predictors therefore depends on the focal country and the date *τ* (e.g., for the 15 European countries and the Hubei province considered in this study, *n* varies between 0 and 15). Predictors *Z*_*i*_ are built from smoothed, scaled and delayed versions of *Y*_*i*_. The smoothing aims at mitigating events that are specific to country *i* (e.g., some eventual delays in the registration of deaths at week-end and the relatively large randomness of increments when the cumulative number of deaths is relatively low). The scaling aims at homogenizing population sizes with the focal country 0. The delay measures the duration between the date *τ* and the anterior date when the predictor *Z*_*i*_ reached the value *Y*_0_(*τ*) (i.e., the advance of country *i* over country 0).

### Modelling the evolution of the cumulative number of deaths in the focal country

The daily increments, say *N*(*t*) = *Y*_0_(*t*)−*Y*_0_(*t*−1), of the cumulative number of deaths in the focal country are considered as random variables drawn under mixtures of negative-binomial distributions whose means are the increments of the competing predictors:

$$N(t)∼\sum_{i=1}^{n}‍p_{i}NB(Z_{i}(t)-Z_{i}(t-1),\eta_{i}),$$

where *p*_*i*_ is the mixture probability describing the relative contribution of data from country *i* to the prediction of the increments in the focal country. Using data at dates *t*≤*τ*, we apply an estimation approach grounded on a weighted penalized likelihood to estimate *p*_*i*_ as well as the dispersion parameters *η*_*i*_ measuring how much the trajectory of the focal country is apart from the trajectory in country *i*. The method is detailed and discussed in S1 File in S1 Data.

Once the model is fitted, it allows (i) the comparison of mortality dynamics between the focal country and the ahead-of-time countries via the mixture probabilities, and (ii) the forecast of the mortality trajectory of the focal country. Instead of selecting a single predictor, the mixture approach enables a probabilistic understanding of relevant predictors. Moreover, the temporal horizon of the forecast depends on the advance of relevant predicting countries. For a predicting country that is *m* days ahead of time, the forecast horizon is *m* days. The longer the targeted temporal horizon, the fewer the available predictors. We stop the forecast at the date when ∑*p*_*i*_<0.5, i.e., when the remaining predictors explain less than 50% of the mortality dynamics in the focal country.

## Results

### Short-term forecast: Performance and results

**Fig 1:**
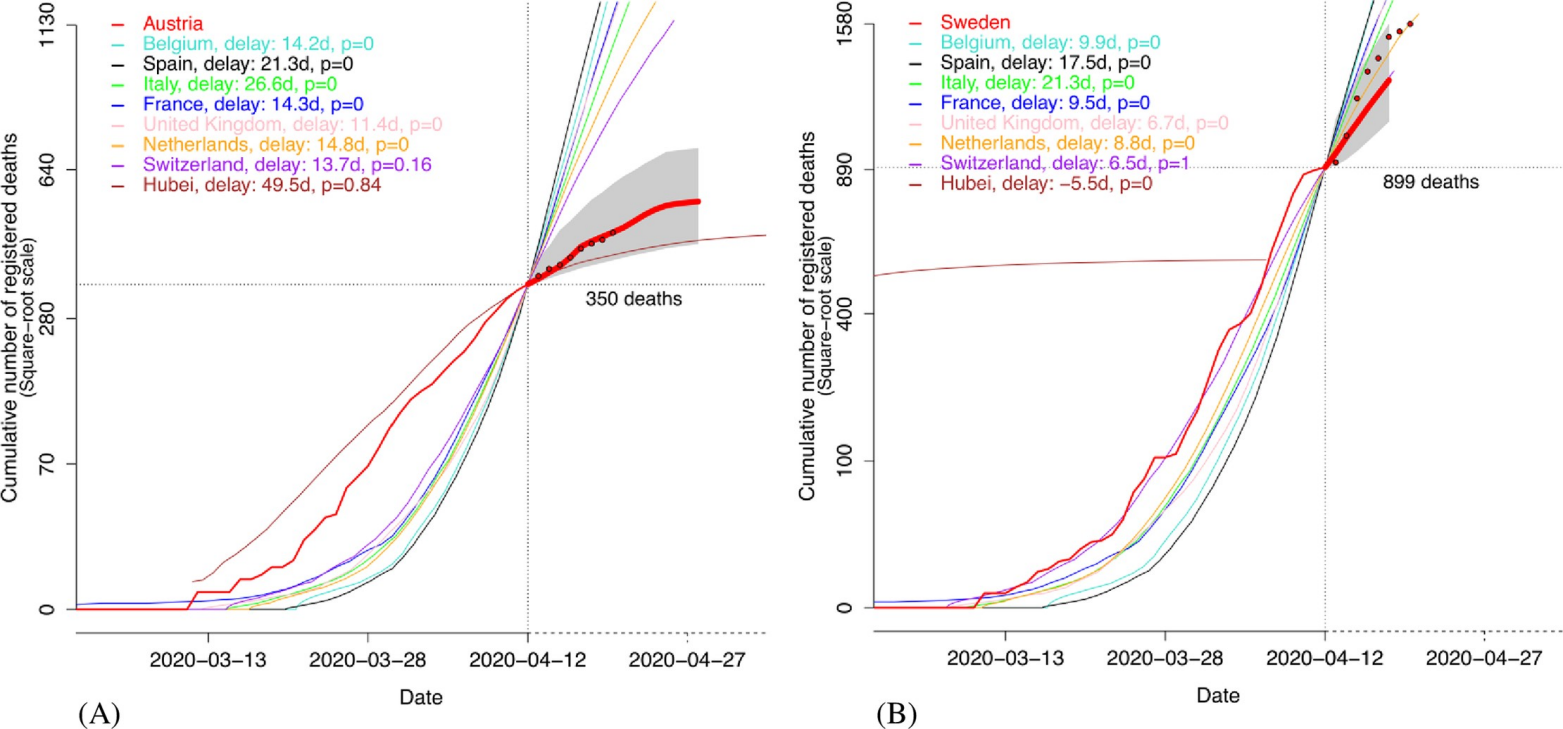
Forecast of the number of deaths from COVID-19 in Austria (A) and Sweden (B) when the last observation is made on April 12, voluntarily ignoring posterior data. Raw mortality data for the focal country are given by the thin red curve up to April 12 and by the red dots afterwards. The estimated cumulative numbers of deaths after April 12 are given by the thick red curve. 95% confidence envelopes are drawn in grey. On April 12, Hubei has a lower death rate than Sweden and can hence not be used as a predictor (thus, the Hubei curve is stopped when it crosses the Swedish curve before April 12).

**Fig 2:**
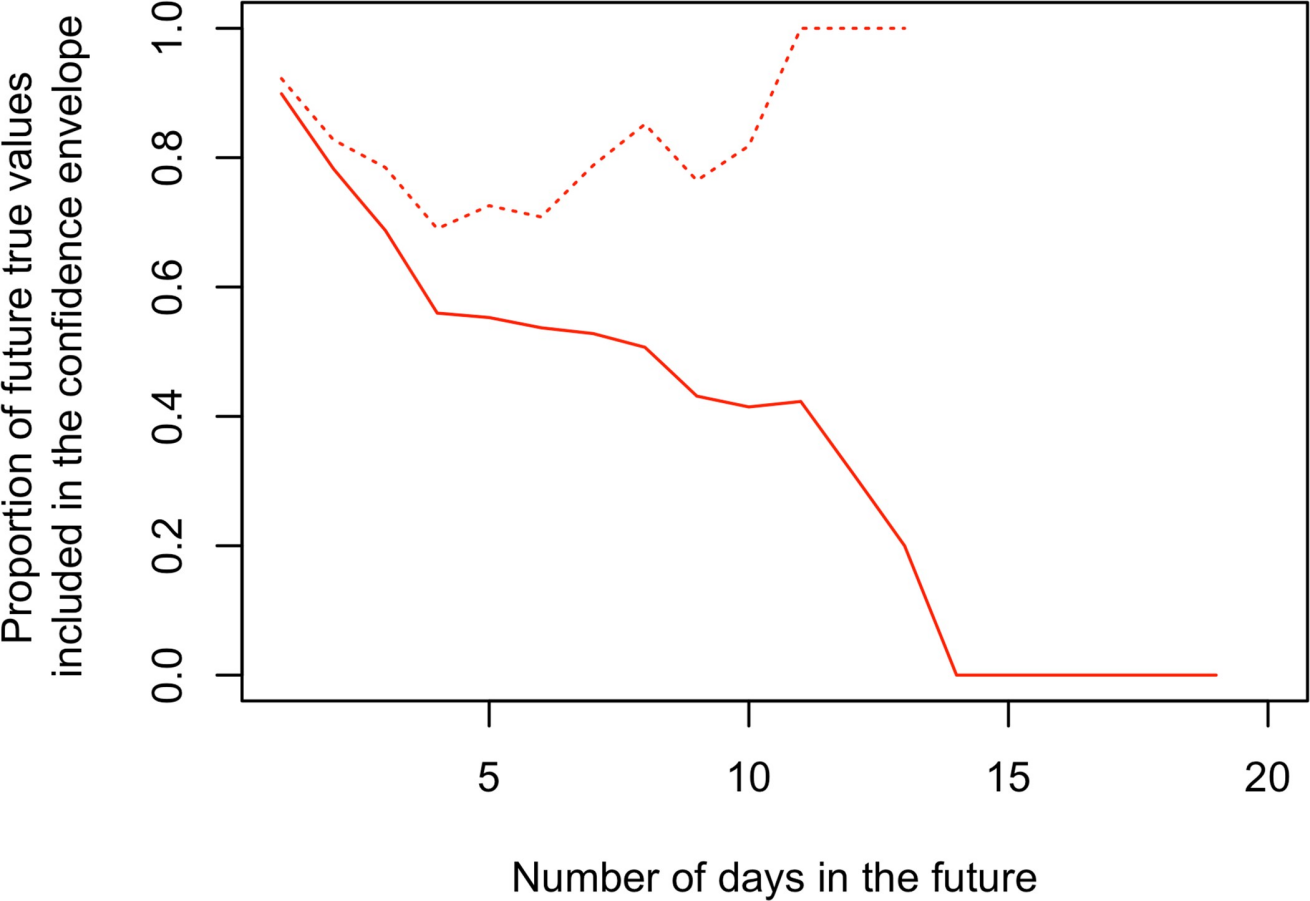
Forecast performance measured as the proportion of true values Y_0_(τ+d), d days after τ, that are in their respective forecast 95%-confidence intervals (CI), i.e., if the red dots in Fig 1 are within the grey confidence envelope also displayed in Fig 1. Solid curve: Proportions calculated by aggregating the eight focal countries, with τ ranging from March 31 to April 19, and using data up to April 20 (to compare the forecast and the actual data). Thus, for any number of days in the future, d, we check for each focal country and for each date τ between March 31 and “April 20 minus d days” if Y_0_(τ+d) is in its respective CI, and we compute the proportion at which this event arises. Dotted curve: Proportions calculated when one only considers situations with at least 250 cumulative deaths at τ.

As explained above, the forecast horizon for a focal country depends on the advance of other countries. To obtain relatively large forecast horizons, we select as predictors the seven European countries offering the longest forecast horizon (i.e., Belgium, France, Italy, Netherlands, Spain, Switzerland, United Kingdom) as well as Hubei, and we forecast the mortality curves of the eight other European countries (i.e., Austria, Denmark, Germany, Ireland, Poland, Portugal, Romania, Sweden). This choice also facilitates comparisons between focal countries since they initially share the same predictors. Qualitatively, forecast performance can be assessed with plots such as those in Fig 1 where we compare, for Austria and Sweden, the forecast made on April 12 (voluntarily ignoring posterior data) with the actual time series after this date. Quantitatively, the average forecast exactness after 10 days is about 80% as soon as the focal country has accumulated at least a few hundreds of deaths; see Fig 2. The performance was computed from the eight focal European countries as the proportion of values *Y*_0_(*t*) observed after *τ* (with *τ* ranging from March 31 to April 19) that are in their respective forecast 95%-confidence intervals (CI). Other forecasts are available in S1-S3 Figs in S1 Data and in the web app http://covid19-forecast.biosp.org.

To get a relative assessment of the short-term forecast performance of our approach, we compare it to a SIRD compartmental model using two distributional assumptions for the observations (that are assumed to follow either a Poisson or a negative-binomial law), and to a log-linear model whose explanatory variables are the scaled mortality dynamics of predicting countries; see S2 File in S1 Data and S4 Fig in S1 Data. Up to the horizon of ten days, only the SIRD—negative-binomial model performs better than our approach in terms of coverage of the true value *Y*_0_(*t*) by the respective CI. Beyond ten days, both the SIRD—negative-binomial and the log-linear models have better coverage of *Y*_0_(*t*). However, the CI obtained with the SIRD—negative-binomial model (resp. the log-linear model) is in average 21 times (resp. 8 times) larger than the CI provided by our approach and therefore leads to a relatively vague prediction. In addition, if the log-linear model is useful for benchmarking, it exploits data from abroad that are synchronous with the mortality dynamics to be predicted and, hence, cannot be used as a forecaster in real situations; see S2 File, Section S2.1 in S1 Data. The low forecast performance beyond 10 days of the mixture model, irrespective of the cumulative number of deaths, possibly results from the lack of relevant predictors that would be sufficiently in advance given the sets of focal and predicting countries that we considered (see Discussion section). Anyway, the validation trial that we designed indicates that the mixture model as it stands is not robust for accurately predicting the number of deaths in a given focal country beyond 10 days (especially when the current cumulative number of deaths is low) and cannot be used in such a setting. To circumvent this limitation, we especially propose in the discussion some alternative choices for the set of predicting countries and the incorporation of a parametric predictor (e.g., grounded on the SIRD model) into the mixture.

### Medium-term and large-scale prediction

**Fig 3:**
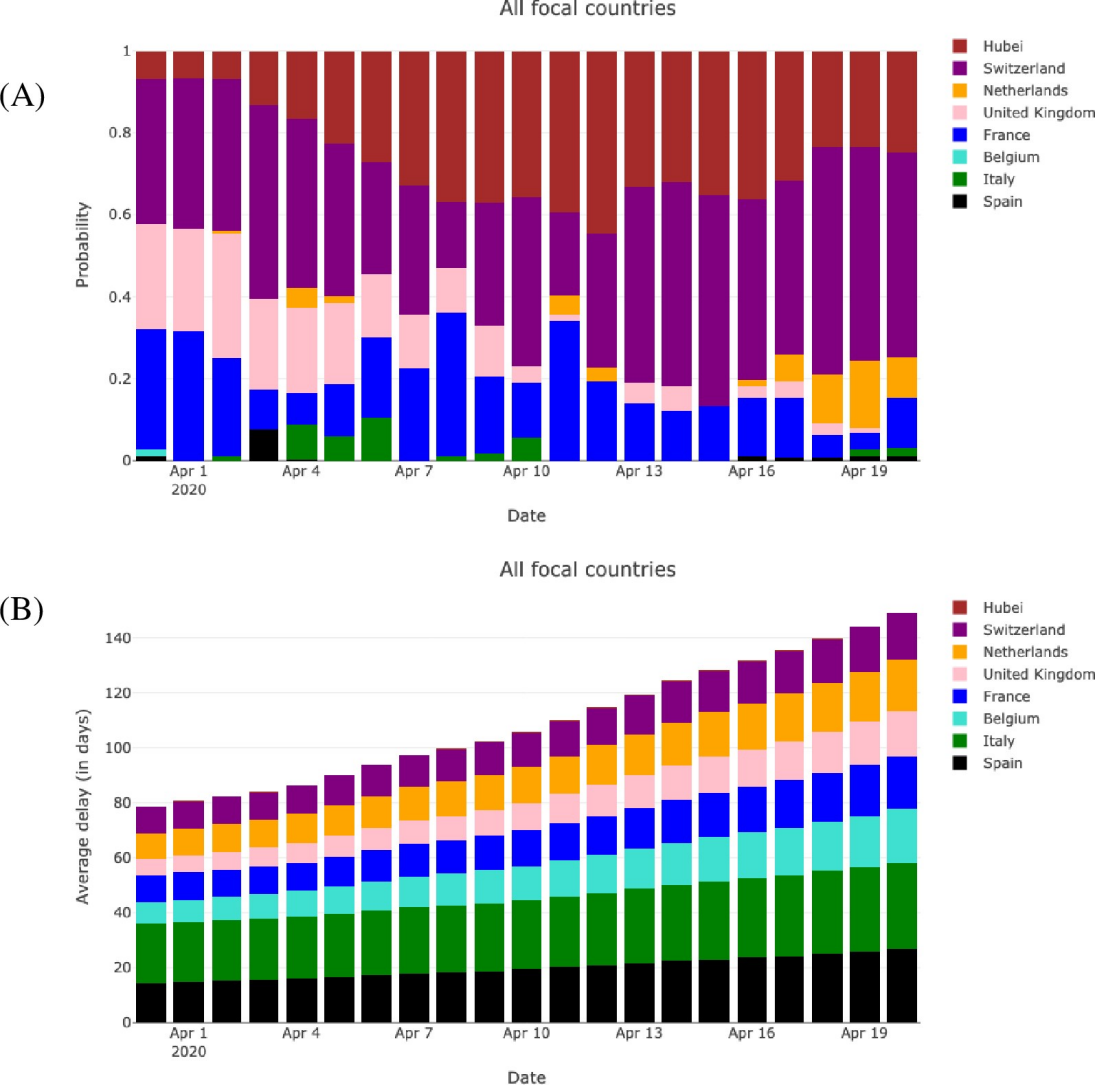
Estimated mixture probabilities (A) and temporal advance of the predicting countries (B) averaged over the eight focal countries and for a date τ of the last observation ranging from March 31 to April 20. The mixture model is fitted to data at each date τ and therefore yields a set of mixture probabilities for each τ and each country (thus, the mixture probabilities for a given focal country may vary across time). The temporal advance is the delay calculated in the construction of the predictors, it is given, e.g., in the legend of Fig 1 for Austria and Sweden on April 12 and is exactly defined in S1 File in S1 Data.

On April 12, the mortality trajectory of Austria follows a mixture of the pasts of Hubei (*p*_*i*_ = 0.84) and Switzerland (*p*_*i*_ = 0.16), whereas the Swedish trajectory follows the past of Switzerland (*p*_*i*_ = 1.00); see Fig 1. The mixture probabilities may vary with the date *τ*, depending on the evolution across time of the COVID-19-induced mortality of the focal and predicting countries. We observe a relative temporal consistency of the mixture probabilities for Austria whereas the mixture probabilities for Sweden are more variable, indicating a larger instability (see S5 Fig in S1 Data). Averaging the mixture probabilities over the eight focal countries shows, overall, that the focal countries slowly tend to increasingly follow the relatively mild trajectories of Hubei and Switzerland (Fig 3A) rather than the more severe trajectories of Belgium, France, Italy, Spain and the United Kingdom (see S6 and S7 Figs in S1 Data enabling a raw comparison of mortality curves). This is confirmed by the increasing average delay between focal countries and predicting countries (Fig 3B), and suggests that, based on data up to mid April, the continuation of the COVID-19 wave across Europe was likely to be mitigated, and not as strong as it was in most of the European countries first impacted by the wave. This conclusion is corroborated by data collected in May: S8 Fig in S1 Data shows that the mortality rate has remained relatively low for most of the second-line countries.

## Discussion

Several modelling approaches have already been proposed to forecast COVID-19 epidemics, most of them relying on standard epidemiological models of the SIR (Susceptible-Infected-Removed) type and their extensions [4–10]. These models generally include compartments corresponding to the dead fraction of the population and can hence be used to predict the temporal evolution of mortality due to COVID-19, in which we are interested here. However, forecasting the epidemic requires to take into account a large amount of factors, including the health system, the control measures, the decision making and even the data collection.

Data-driven approaches involving artificial intelligence-inspired methods [11], coupled SIR-neural network approaches [12] and web-trends data analysis [13] have also been proposed. In these cases, the training data concern past signals in the considered country and therefore do not bring information on processes that only arise when some thresholds in the number of deaths are reached (e.g., saturation of medical structures and lockdown), if the thresholds into question have not been reached yet.

For countries where the outbreak started later than for the first impacted countries, our approach contributes to develop a complementary and twofold vision of forecast. It firstly provides short-term and real-time forecasting that are provided by the dedicated web app http://covid19-forecast.biosp.org. It secondly allows a probabilistic comparison of the mortality dynamics in a focal country to past dynamics in ahead-of-time countries. These past dynamics form real-life models and, given the inertia of COVID-19-like epidemics, they can be combined to design medium-term trends under the settings of mixture modelling.

In our approach, the choice of the mixture components is critical to some extent. The set of real-life predictors that is used may introduce a bias, as observed for Poland for example: none of the predictors that are used have sufficiently mild mortality dynamics in comparison with the Polish mortality dynamics; see S1-S3 Figs in S1 Data. The reasons could result from multiple factors, such as differences in connections with disease epicenters in the world, economies, health care systems, lockdown measures, climatic variables, population sizes and densities. Thus, a preliminary step for the selection of the set of predictors could include such factors. In the manuscript, since our aim was to compare front-line and second-line European countries, we used the same predictors for all the focal countries. However, in the web app, predictors are selected in a different way: they consist of countries with high mortality rates (providing a sort of upper boundary for mortality), of countries with close raw mortality dynamics and of countries with close population sizes. Other criteria could be used, but one must make the balance between these criteria and the availability of predictors that are sufficiently in advance. For instance, on July 5, 2020, the mortality dynamics of Colombia follows a mixture of the dynamics of Bolivia (p = 0.51), US Iowa (p = 0.25), US Mississippi (p = 0.18) and Netherlands (p = 0.06) despite apparent differences in economies and health care systems in these 5 countries (see the corresponding capture of the web app in S9 Fig in S1 Data). In this example, it would not be necessarily relevant to *a priori* use economic and health care system criteria to preliminary choose potential predictors.

An interesting perspective for handling cases where the mixture of the predicting-countries dynamics does not achieve a satisfactory goodness-of-fit, whatever the chosen set of predicting countries, consists in adding a parametric predictor to the set of predictors in the mixture model. We present this approach in S1 File (Section S1.4) in S1 Data using a parametric predictor grounded on the SIRD model introduced above in the forecast performance comparison, and we apply it in S10 and S11 Figs in S1 Data for Sweden and Poland. In these two cases, the parametric predictor gets a relatively high probability (0.70 for Sweden, 0.94 for Poland), but this is not always the case as shown in S12 Fig in S1 Data for Kyrgyzstan where the parametric predictor has a zero probability. This approach may be relevant for both extending the forecast horizon of the mixture model and improving its forecast performance, especially beyond the horizon of 10 days. Indeed, the prediction resulting from the parametric predictor is not limited in time in contrast to those furnished by the predicting-countries dynamics, and the incorporation of the parametric predictor is expected to bring a solution to cases where none of the predicting countries are adequate as discussed in the previous paragraph. Other perspectives for improving the forecast performance are presented in the technical discussion included in S1 File (Section S1.5) in S1 Data.

In further work, our approach could be adapted for screening the impact of vaccination or therapy on COVID-19-induced mortality with country-to-countries comparisons. Moreover, it would be interesting to unravel the relationship between the mixture probabilities and some potentially explanatory factors: Can we identify the same characteristics concerning population structures, health care systems, control measures, etc., in the focal countries and their most likely predicting countries as defined by the mixture probabilities? We could also extend the application of our approach at the intra-country resolution to compare and monitor, in real-time, mortality dynamics across regions, and disentangle the consequences of heterogeneities in, e.g., social structures and control-strategy implementation.

## Supporting information

S1 Data (ZIP)
