# Supplementary material for: COVID-19 mortality dynamics: The future modelled as a (mixture of) past(s)
Source: PLoS One. 2020 Sep 11;15(9):e0238410. doi: 10.1371/journal.pone.0238410 (PMC7485826; doi:10.1371/journal.pone.0238410)
Supplement: S1 Data — (ZIP) [file pone.0238410.s001.zip › melange-Suppl_S1file.pdf]

S1 Supporting Text – Details of the method

S1.1 Mixture model

Let Y_i $(i = 0, \dots, n)$ denote non-decreasing temporal counting processes over $\mathbb{Z}$ observed up to time τ corresponding to different observation units with varying sizes. The sizes of the observation units are known scalar values denoted by $s_i > 0$. Typically, $Y_i(t)$ denote the cumulative number of deaths due to a disease in population i at time t, and s_i is the population size. Y_0 is a process to be forecast beyond τ. The n processes Y_i $(i > 0)$, which are supposed to be *ahead in time* with respect to Y_0, will be used to build putative predictors for Y_0.

The increments of the process Y_0 are independently drawn from mixtures of negative-binomial distributions whose means are the increments of competing predictors:

$$\begin{aligned} Y_0(t) &= Y_0(t-1) + N(t) \\ N(t) &\underset{\text{indep.}}{\sim} \sum_{i=1}^{n} p_i \mathcal{NB}\Big(Z_i(t) - Z_i(t-1), \eta_i\Big), \end{aligned} \tag{1}$$

where p_i are the mixture probabilities, η_i are the dispersion parameters of the negative-binomial distributions, and Z_i are the predictors built from smoothed, scaled and delayed versions of Y_i $(i = 1, \dots, n)$; note that the model for Y_0 is defined conditionally on the set of predictors Z_i (thus, the subscript 'indep.', under the symbol $\sim$ denoting *equality in distribution*, means the conditional independence of the $N(t)$s when t varies, given the predictors Z_i). Namely, Z_i satisfies, for all $t \in \mathbb{R}$:

$$\begin{aligned} Z_i(t) &= \bar{Y}_{0i}(t - \delta_i) \\ \bar{Y}_{0i}(t) &= \frac{s_0}{s_i}(\mathcal{L}\bar{Y}_i)(t) \\ \bar{Y}_i(u) &= \frac{1}{2k_u + 1} \sum_{t'=u-k_u}^{u+k_u} Y_i(t'), \quad \forall u \in \mathbb{Z}, \\ \delta_i &= \tau - \min\{t \in \mathbb{R} : \bar{Y}_{0i}(t) = Y_0(\tau)\}, \end{aligned} \tag{2}$$

where $\mathcal{L}$ is the linear-interpolation operator (i.e., $\bar{Y}_{0i}$ is a continuous-time version of $\bar{Y}_i$ that exactly coincides with $\bar{Y}_i$ at the observation times of this (discrete-time) process and that is linear between the observation times), $k_u = \min\{k, \tau - u\}$ is the local smoothing bandwidth, and $k \in \mathbb{N}$ is the smoothing parameter (if $k = 0$, there is no smoothing: $\bar{Y}_i \equiv Y_i$). The process $\bar{Y}_i$ defined over $\mathbb{Z}$ is obtained by smoothing Y_i with a symmetric local mean based on the varying temporal bandwidth k_t, with adaptation to ensure that $\bar{Y}_i(\tau) = Y_i(\tau)$, i.e. the last observed value of Y_i, and hence avoid border effects due to smoothing. Thus, $\bar{Y}_i$ is a moving average operator applied to Y_i. The process $\bar{Y}_{0i}$ defined over $\mathbb{R}$ is obtained by linearly interpolating $\bar{Y}_i$ and by scaling it with the factor s_0/s_i to delete the heterogeneity in the sizes of the observation units. Note that if $\bar{Y}_{0i}$ is defined over $\mathbb{R}$, it can be only computed up to time τ from observed data. The predictor Z_i is built from a smoothed version of Y_i to mitigate events that are specific to observation unit i (and that are not expected to be representative of what is going on in observation unit 0). The delay δ_i is introduced to measure how much the observation unit i is ahead in time with respect to the observation unit 0, and to correct the non-delayed predictor $\bar{Y}_{0i}$ accordingly. In the expression of δ_i, $\min\{t \in \mathbb{R} : \bar{Y}_{0i}(t) = Y_0(\tau)\}$ is the time at which the process $\bar{Y}_{0i}$ reaches the level $Y_0(\tau)$; δ_i is therefore the duration between this time and τ. Figure S0 illustrates the computation of δ_i. Thus, in practice, the predictor Z_i can be computed up to the temporal horizon $\tau + \delta_i$, which depends on the calculated advance of Y_i over Y_0.

The condition $Y_i(\tau)/s_i > Y_0(\tau)/s_0$ (which can be easily checked../ in practice directly from raw data) ensures that Y_i is *ahead in time* with respect to Y_0 and can be used to build a predictor. The way we define *a country ahead in time* is irrespective of the actual date at which the epidemics in this country and the focal country were initiated. In contrast, our approach relies on the idea that if a country i is a relevant predictor, the scaled process Y_i/s_i (or more exactly its smoothed version) was equal to $Y_0(\tau)$ in the past of τ (namely at time $\tau - \delta_i$) *and* the curvature of the mortality dynamics 'Y_0 before τ' and 'Y_i/s_i before $\tau - \delta_i$' are similar.

Note that Z_i (and $\bar{Y}_{0i}$) are non-decreasing temporal processes over $\mathbb{R}$ and, therefore, the means of the negative-binomial distributions in Equation (1) are non-negative.

S1.2 Weighted penalized likelihood

Suppose that the discrete time processes Y_i $(i = 0, \dots, n)$ are observed between time τ_0 and τ. Our aim is to infer the mixture probabilities $\mathbf{p} = (p_1, \dots, p_n)$ and the dispersion parameters $\boldsymbol{\eta} = (\eta_1, \dots, \eta_n)$ based on $\mathbf{Y} = \{Y_i(t) : i = 0, \dots, n, t = \tau_0, \dots, \tau\}$. Using Equations (1) and (2), the likelihood satisfies:

$$L(\mathbf{p} \mid \mathbf{Y}) = \prod_{t=\tau_0+1}^{\tau} \sum_{i=1}^{n} p_i f_{0it}, \tag{3}$$

where f_{0it} is the probability that a variable following the negative-binomial distribution with mean $Z_i(t) - Z_i(t-1)$ and dispersion parameter η_i equals $Y_0(t) - Y_0(t-1)$ (the variance of this variable is $(Z_i(t) - Z_i(t-1)) + (Z_i(t) - Z_i(t-1))^2/\eta_i$; the lower the value of η_i, the larger the dispersion).

The likelihood is not directly used to estimate $\mathbf{p}$ and $\boldsymbol{\eta}$, but is first weighted and penalized. The weighting allows the estimation to depend more on the last states of the response process Y_0 and the predictors than on their initial states (e.g., to avoid an irrelevant dependence of the estimation on relatively ancient fluctuations that are likely poorly related to the current variations). The penalization is introduced to improve the prediction accuracy and interpretability of mixture probabilities by penalizing predictors that are unlikely at least on some fragments of the observation window.

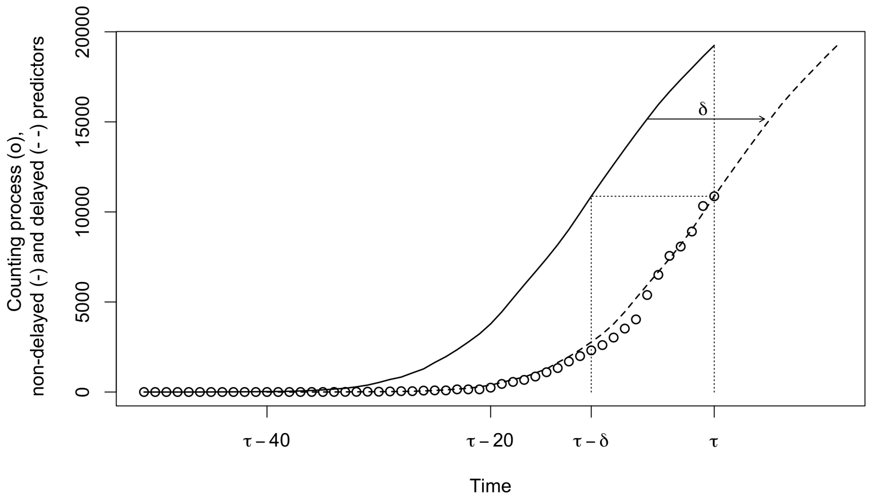

Figure S0: Schematic representation of the delaying processing applied to predictors. Circles: counting process Y_0; Continuous line: non-delayed predictor $\bar{Y}_{0i}$; Dashed line: delayed predictor Z_i (i.e. δ-translation of $\bar{Y}_{0i}$ along the time-axis, where $\delta = \delta_i$ in Equation (2)).

Thus, we propose the following weighted penalized log-likelihood for estimating $\mathbf{p}$:

$$\begin{aligned} \tilde{l}_\lambda(\mathbf{p}, \boldsymbol{\eta} \mid \mathbf{Y}) &= \left(\sum_{t=\tau_0+1}^{\tau} w_{\tau-t} \log \sum_{i=1}^{n} p_i f_{0it} \right) - \left(\lambda \sum_{t=\tau_0+1}^{\tau} \sum_{i=1}^{n} p_i \mathbb{1}(f_{0it} = 0) \right) \\ w_{\tau-t} &= \left(\frac{t-\tau_0}{\tau-\tau_0} \right)^2, \end{aligned} \tag{4}$$

where $\lambda \geq 0$ and $\mathbb{1}$ is the indicator function. The squared shape of $w_{\tau-t}$ can obviously be substituted, depending on the context, for instance by a linear shape. As we can see in Equation (4), a predictor i is said to be "unlikely at least on some fragments of the observation window" when the probability f_{0it} to observe $Y_0(t) - Y_0(t-1)$ under the negative-binomial distribution with mean $Z_i(t) - Z_i(t-1)$ and dispersion parameter η_i is zero for some dates t. Thus, the predictor i is penalized by $\lambda \times p_i$ times the frequency (across time) of the event $f_{0it} = 0$. Hence, a large frequency of $f_{0it} = 0$ for fixed i leads the algorithm to favor a small value for p_i.

S1.3 Estimation and forecast

For fixed τ_0, k and λ, the parameter vectors $\mathbf{p}$ and $\boldsymbol{\eta}$ can be estimated by maximizing $\tilde{l}_\lambda(\mathbf{p}, \boldsymbol{\eta}, \mid \mathbf{Y})$ given by Equation (4) with a constrained BFGS quasi-Newton method (Byrd et al., 1995). This algorithm easily handles positiveness of parameters. The constraint $\sum_{i=1}^n p_i = 1$ was handled by introducing $q_i \in [0, \infty)$, $i = 1, \ldots, n$, such that $q_i / \sum_{i'=1}^n q_{i'} = p_i$, fixing one of the q_is to the value 1 and maximizing $\tilde{l}_\lambda(\mathbf{p}, \boldsymbol{\eta}, \mid \mathbf{Y})$ with respect to the other q_is and $\boldsymbol{\eta}$. Let us now describe how τ_0, k and λ are specified. The first observation time τ_0 and the maximal bandwidth k are considered as given for the analysis of COVID-19 data: we use $\tau_0 = \tau - 30$ days allowing our analysis to handle the inertia of the epidemics at country level and $k = 3$ days resulting in rather smooth predictors. The penalization parameter λ, whose value cannot be intuitively fixed unlike τ_0 and k, is calibrated by minimizing the mean squared error between $(Y_0(\tau-2), Y_0(\tau-1), Y_0(\tau))$ and its mixture-based prediction obtained by using only data up to time $\tau - 3$. The mixture-based prediction of $Y_0(t)$ for t beyond the last observation date τ is simply its expectation satisfying:

$$\mathbb{E}(Y_0(t)) = Y_0(\tau) + \sum_{t'=\tau+1}^{t} \sum_{i=1}^{n} p_i (Z_i(t') - Z_i(t'-1)). \tag{5}$$

Thus, λ is fixed at the value $\hat{\lambda}$ satisfying:

$$\hat{\lambda} = \underset{\lambda \geq 0}{\operatorname{argmin}} \frac{1}{3} \sum_{t'=\tau-2}^{\tau} \left(\hat{\mathbb{E}}_{1:(\tau-3),\lambda}(Y_0(t')) - Y_0(t') \right)^2$$

where $\hat{\mathbb{E}}_{1:(\tau-3),\lambda}(Y_0(t'))$ is derived from Equation (5), in which τ is replaced by $\tau - 3$ and p_i are replaced by their estimates $\hat{p}_i(\lambda)$ maximizing $\tilde{l}_\lambda(\mathbf{p}, \boldsymbol{\eta} \mid \mathbf{Y}_{1:(\tau-3)})$, itself derived from Equation (4) where $\mathbf{Y}_{1:(\tau-3)}$ gathers data up to time $\tau - 3$ and replaces $\mathbf{Y}$.

Then, estimates $\hat{\mathbf{p}}$ and $\hat{\boldsymbol{\eta}}$ of $\mathbf{p}$ and $\boldsymbol{\eta}$ are obtained by maximizing $\tilde{l}_{\hat{\lambda}}(\mathbf{p}, \boldsymbol{\eta} \mid \mathbf{Y})$ and the dynamics of Y_0 beyond τ is forecast by simulating Model (1) where one plugs in $\hat{\mathbf{p}}$ and $\hat{\boldsymbol{\eta}}$ and by drawing, for instance, 95%-confidence envelopes. The forecast horizon of Y_0 depends on how much each predicting countries is ahead in time. E.g., for a predicting country that is m days ahead in time, the forecast horizon is m days. Thus, when time goes on beyond τ, there are less and less available predictors. In our analyses, we stop the forecast at the date when available predictors correspond to a sum of probabilities $\hat{p}_i$ less than 0.5. An alternative of the forecast can be drawn by assuming that, for each simulation, the trajectory of the epidemic follows a single predictor sampled with respect to the mixture probabilities. This approach generally leads to wider 95%-confidence envelopes. The threshold 0.5 could also be avoided by re-estimating the mixture probabilities when a predictor is not available anymore (instead of just re-weighting the mixture probabilities of predictors that are still available). Thus, we could extend the forecast horizon.

S1.4 Adding a parametric predictor to the mixture

To handle cases where the mixture of the predicting-countries dynamics does not achieve a satisfactory goodness-of-fit, a parametric predictor can be added to the list of predictors. For instance, we propose to include the following $(n+1)$th predictor in the mixture:

$$Y_{n+1}(t) = Y_{n+1}(\tau_0) + c_0(e^{c_1\,(t-\tau_0)} - 1),$$

where $\tau_0 = \tau - 30$ days like in Section S1.3, and c_0 and c_1 are real coefficients to be estimated beforehand. This parametric form results from a SIRD compartmental model (susceptible - infectious - recovered - dead) presented in Supporting Text S2. Parameters c_0 and c_1 are preliminarily estimated by minimizing with respect to c_1 the following sum of least squares $\sum_{t=\tau_0}^{\tau}(Y_0(t) - Y_{n+1}(t))^2$ under the constraints $Y_{n+1}(\tau_0) = Y_0(\tau_0)$ and $Y_{n+1}(\tau) = Y_0(\tau)$. These constraints mean that the parametric predictor coincides with Y_0 at times τ_0 and τ and imply that c_0 is deterministically defined by the following expression depending on c_1:

$$c_0 = (Y_0(\tau) - Y_0(\tau_0))/(e^{c_1(\tau-\tau_0)} - 1).$$

Supporting Figures S10 and S11 show, for Sweden and Poland, respectively, the forecast obtained either without or with the parametric predictor proposed above for data used up to July 5, 2020. In these two cases, the parametric predictor has a relatively high probability. This is not always the case as shown in Figure S12 for Kyrgyzstan where the parametric predictor has a zero probability.

S1.5 Technical discussion

In our approach, the quality of data is paramount, as is the case with most approaches strongly relying on data. However, some technical choices underlying our method (in particular the smoothing of predictors and the over-dispersed distribution for the daily increments in the number of deaths) make our approach robust to some extent. For instance, Swedish data show a decrease in the number of recorded daily deaths each week-end, but its mortality trajectory is nonetheless relatively adequately forecast. Despite the relative robustness of our approach, its performance would be improved by grounding it on corrected data (e.g., by re-allocating some deaths recorded on Mondays and Tuesdays in Sweden to the preceding weekend, and we could correct French data anterior to April 1st, before which deaths in nursing homes were not recorded). Correcting data would be particularly crucial for countries that may underestimate COVID-19-induced deaths. It must be however noted that our simple approach applied to raw data can be exploited to detect countries with *extreme* trajectories (i.e., very mild mortality dynamics), resulting either from bias in data or from particularly efficient ways of mitigating the COVID-19 wave. Identifying the former case can be beneficial to obtain a better assessment of COVID-19 sanitary impact in the country of interest. Identifying the latter case can be beneficial for other countries to improve their control strategies by taking as a model the strategy of the country with extreme trajectory.

In our analysis, we incorporated eight predictors in the mixture model (seven *front-line* European countries plus Hubei). Having at our disposal more predictors would be obviously beneficial for our analyses, in particular intermediate predictors between those built from Switzerland and Hubei data, and even flatter predictors than the one built from Hubei data, e.g., for handling the apparently very mild mortality curve of Poland. The incorporation in the mixture model of mortality curves corresponding to other well-known diseases deserves to be investigated. These additional curves could be viewed as benchmarks complementing the available predictors for COVID-19. From a technical viewpoint, multiplying the number of mixture components could result in estimation difficulties if one only relies on the quasi-Newton algorithm that we used for maximizing the penalized weighted likelihood. Alternatively, parameter estimation could be carried out using the Expectation Maximization (EM) algorithm (McLachlan and Peel, 2000) or adopting a Bayesian framework, with Markov Chain Monte Carlo (MCMC) techniques (Robert and Casella, 2013).

In our mixture model approach, we used the negative-binomial distribution because the increments in mortality are counting variables and because we observed a relatively large dispersion with respect to the predictors. The Poisson distribution could not cope with this over-dispersion and led therefore to poor results in terms of forecast performance. It must be however noted that in a hypothetical case without overdispersion, the estimation of dispersal parameters $\boldsymbol{\eta}$ in the negative-binomial distributions would lead to identifiability issues. Other distributional hypotheses could be made, e.g. discrete versions of the exponential and gamma distributions defined over $\mathbb{N}$, for which EM algorithms are well suited in a mixture-model context.

The negative-binomial assumption leads to relatively large confidence envelopes in cases where the cumulative number of death at time τ (i.e., the time at which the forecast is made) is low and none of the used predictors is particularly close from the focal dynamics; see Supporting Figures S1-S3. Nevertheless, providing large confidence envelopes when information in data are relatively low and any single predictor does not particularly fit the focal dynamics is in fact a fair feature of our approach.

We used a linear interpolation in Equation (2) because of its simplicity. The use of splines could be investigated to evaluate whether this refinement improves forecast performance. Forecast performance could also be improved by: 1. optimizing the smoothing parameter or even by changing the form of the smoother that we used (e.g. local polynomial smoother, kernel smoother with a continuous kernel, etc.); 2. estimating the delay between focal and predicting countries jointly with the mixture weights and the dispersion parameters instead of calculating it *a priori* (note that if a parametric predictor is added to the mixture as proposed in Section S1.4, its parameters could also be jointly estimated with the mixture weights); 3. refining the penalization and the weighting introduced in Equation (4); 4. transforming the mixture model into a regression model and applying, for example, an elastic net penalization to reduce the number of likely predictors; 5. incorporating multiple-orders auto-regressive terms in the model of Y_0 to better account for temporal dependencies, but the auto-regressive terms should certainly be modified depending on the stage of the epidemics (increase stage, decrease stage and plateau); 6. incorporating additional covariates to account for temporal characteristics that are specific to the focal country (e.g., a change in the national control strategy); 7. considering an other scaling than the scaling of cumulative mortality by the population size. Concerning point 7, we consider in the application countries with very different population sizes (from 5 to 83 millions) and, based on initial analyses, we observed a higher

forecast performance when we used the scaling by the population size, i.e. when we used the death rates, than when we did not introduced this scaling, i.e. when we used the number of deaths. However, other scalings could be proposed, e.g., the ratio of the sizes of the populations over 65 years old (we implemented this option in the web app).

References

Byrd, R. H., P. Lu, J. Nocedal, and C. Zhu (1995). A limited memory algorithm for bound constrained optimization. *SIAM Journal on scientific computing 16*, 1190–1208.

McLachlan, G. and D. Peel (2000). *Finite Mixture Models*. John Wiley & Sons: New York.

Robert, C. and G. Casella (2013). *Monte Carlo statistical methods*. Springer Science & Business Media.
