# Supplementary material for: COVID-19 mortality dynamics: The future modelled as a (mixture of) past(s)
Source: PLoS One. 2020 Sep 11;15(9):e0238410. doi: 10.1371/journal.pone.0238410 (PMC7485826; doi:10.1371/journal.pone.0238410)

Figure S2. Forecast of the number of deaths from COVID-19 in Austria, Denmark, Germany, Ireland, Poland, Portugal, Romania and Sweden when the last observation is made on April 6, voluntarily ignoring posterior data. Raw mortality data for the focal country are given by the thin red solid curve up to April 6 and by the red dots afterwards. The estimated cumulative number of deaths after April 6 is given by the thick red solid curve. 95% confidence envelopes are drawn in grey.

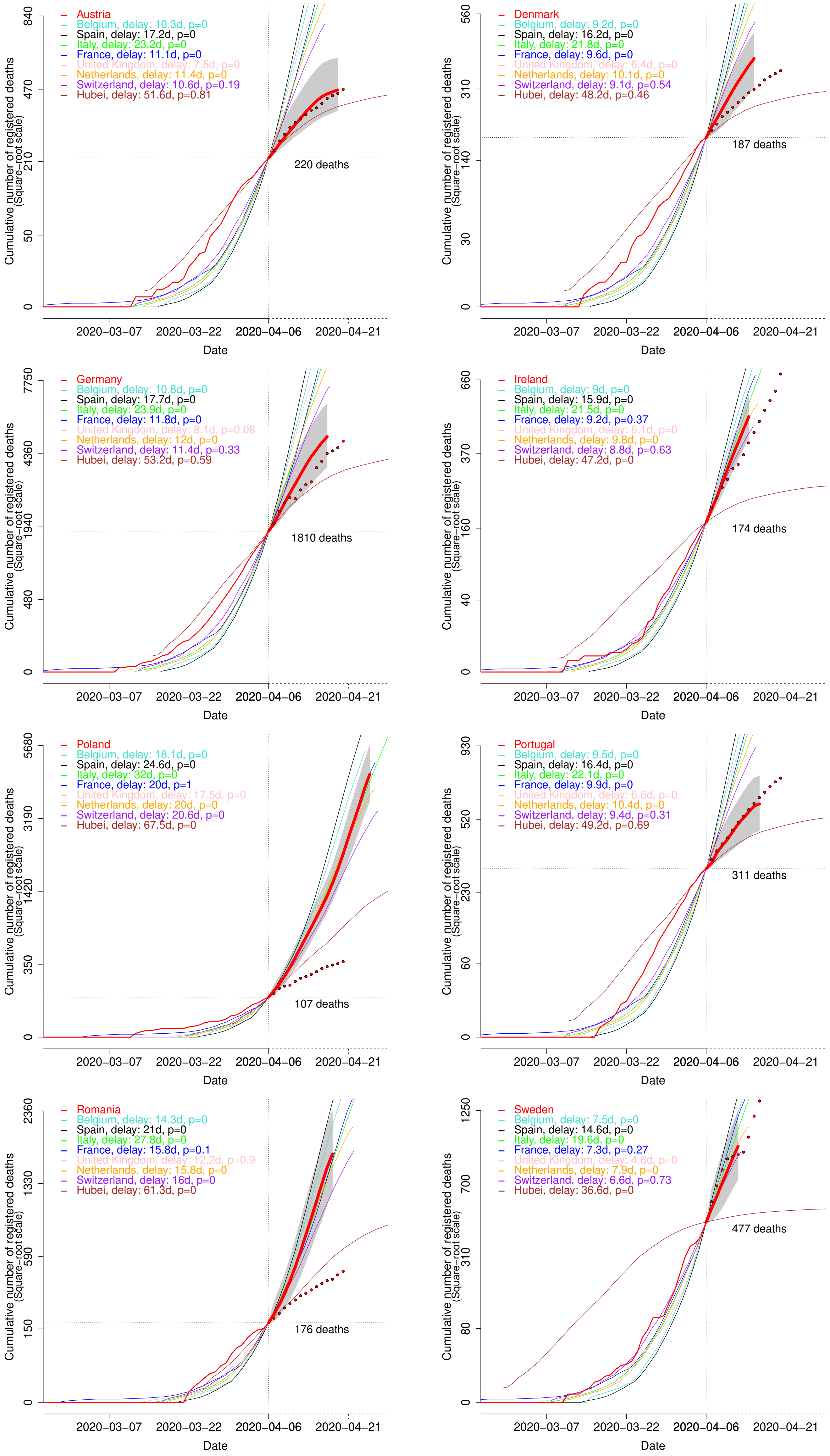

Supplement: S1 Data — (ZIP) [file pone.0238410.s001.zip › melange-Suppl_S2fig.pdf]
