# Supplementary material for: COVID-19 mortality dynamics: The future modelled as a (mixture of) past(s)
Source: PLoS One. 2020 Sep 11;15(9):e0238410. doi: 10.1371/journal.pone.0238410 (PMC7485826; doi:10.1371/journal.pone.0238410)
Supplement: S1 Data — (ZIP) [file pone.0238410.s001.zip › melange-Suppl_S2file.pdf]

S2 Supporting Text – Alternative models

We compared the performance of the mixture model in terms of forecast accuracy with two relatively simple models: a SIRD compartmental model (susceptible - infectious - recovered - dead) only fitted to data collected from the population of the focal country, and a log-linear model whose explanatory variables are the scaled mortality dynamics of predicting countries.

S2.1 SIRD model

Using the same notation as in Supporting Text S1, the expectation of the cumulative number of deaths in the focal population satisfies, under the SIRD model that we consider:

$$\mathbb{E}(Y_0(t)) = Y_0(\tau_0) + c_0(e^{c_1\,(t-\tau_0)} - 1), \tag{1}$$

where $\tau_0 = \tau - 30$ days like in Section S1.3, and c_0 and c_1 are real coefficients to be estimated. Equation (1) is derived from a continuous-time compartmental model with susceptible, infectious, recovered and dead individuals that can be described as follows. Assume that the effective reproduction number R_e can be considered as being constant over a temporal window $\tau \pm \Delta$ (meaning that the contact rate and the number of susceptible individuals are approximately stable during this period of time). Then, during this phase, the number of infectious individuals follows an exponential dynamic (either increasing if $R_e > 1$ or decreasing otherwise): $I(t) = I_0\,e^{\alpha\,t}$. The number of deaths $D(t)$ depends on the number of infectious during the past days. More precisely, the time-derivative of $D(t)$ is assumed to satisfy $D'(t) = \gamma\,I(t-\beta)$, for some time lag β. Thus $D'(t) = \gamma\,I_0\,e^{\alpha\,(t-\beta)}$ and, by integrating D' over time between τ_0 and t, $D(t) = D(\tau_0) + c_0(e^{c_1\,(t-\tau_0)} - 1)$ where c_0 and c_1 are real constant coefficients. This formula is then used in discrete-time to obtain Equation (1).

The SIRD model is fitted to mortality data collected from the population of interest in the framework of generalized non-linear models (Vonesh and Chinchilli, 1996), using either a Poisson law or a negative-binomial law for the distributional assumption, i.e.:

$$Y_0(t) \mid Y_0(\tau_0) \;\sim\; \mathcal{P}\big(Y_0(\tau_0) + c_0(e^{c_1\,(t-\tau_0)} - 1)\big), \tag{2}$$

or

$$Y_0(t) \mid Y_0(\tau_0) \;\sim\; \mathcal{NB}\big(Y_0(\tau_0) + c_0(e^{c_1\,(t-\tau_0)} - 1), \theta\big), \tag{3}$$

where the negative-binomial distribution is parameterized by the mean given by Equation (1) and the dispersion parameter denoted by $\theta > 0$. Thus, c_0, c_1 and θ are estimated by maximizing the log-likelihood corresponding to each distributional assumption and to data from τ_0 to τ. The estimates are then simply plugged in (2) or (3) to forecast $Y_0(t)$ for $t > \tau$, i.e. to provide the mean and confidence intervals of $Y_0(t)$.

S2.2 Log-linear model

Using the same notation as in Supporting Text S1, we assume that Y_0 is generated from the simple log-linear model defined by the following equation:

$$\log(Y_0(t) + 1) = \beta_0 + \sum_{i=1}^{n} \beta_i \log(Y_{0i}(t) + 1) + \varepsilon(t), \tag{4}$$

where the βs are unknown real coefficients and ε is a centered Gaussian noise with fixed but unknown variance. This model can be simply fitted to data with a function allowing to fit linear regressions. It is used here as a benchmark using data from abroad, like the mixture model. However, in real situations, it cannot be used to forecast Y_0 since the scaled mortality dynamics Y_{0i} are used in a synchronous manner (whereas they are used by introducing a delay in the mixture model).

S2.3 Forecast performance comparison

Supporting Figure S4 provides forecast performance for the SIRD model with Poisson distribution (2), the SIRD model with negative-binomial distribution (3), the log-linear model (4) and our mixture model. The forecast performance is calculated from real data: it is measured as the proportion of true values $Y_0(\tau + d)$, d days after τ, that are in the corresponding forecast 95%-confidence intervals obtained from each model. The proportions are calculated by aggregating the eight focal countries, with τ ranging from March 31 to April 19, and using data up to April 20 (to compare the forecast and the actual data). This approach, where we voluntarily ignore the future of the dynamics and we check whether this future is correctly forecast, allows us to consider a real situation where data have not been generated by the models in competition (hence, we assess forecast performance of the different models without favoring one of them).

Up to the temporal horizon of 10 days, the mixture model performs better than the SIRD–Poisson model and the log-linear model but, at first sight, is less efficient than the SIRD–negative-binomial model (beyond 10 days, the assessment of the forecast performance is less reliable because it is based on a lower number of repetitions; this is particularly true in the case of the mixture model, for which the temporal horizon depends on the availability of predictors that are sufficiently in advance). However, in average, the 95%-confidence intervals (CI) provided by the SIRD–negative-binomial model are 21 times wider than those provided by the mixture model, the 95%-CI provided by SIRD–Poisson model are 1.1 times smaller than those given by the mixture model, and the 95%-CI provided by the log-linear model are 8.1 times wider than those given by the mixture model. Given the relative lengths of CI, we consider that the mixture model performs relatively well up to 10 days. This performance however depends on the adequacy of the predicting countries that are used for each focal country, and may either increase or decrease accordingly.

References

Vonesh, E. and V. M. Chinchilli (1996). *Linear and nonlinear models for the analysis of repeated measurements.* CRC press.
