# Supplementary material for: COVID-19 mortality dynamics: The future modelled as a (mixture of) past(s)
Source: PLoS One. 2020 Sep 11;15(9):e0238410. doi: 10.1371/journal.pone.0238410 (PMC7485826; doi:10.1371/journal.pone.0238410)

Figure S4. Forecast performance obtained with the mixture model (red), the SIRD–Poisson model (blue), the SIRD–negative-binomial model (turquoise) and the log-linear model (black). The performance is measured as the proportion of true values $Y_0(\tau + d)$, d days after τ, that are in the corresponding forecast confidence intervals obtained from each model. Solid curve: Proportions calculated by aggregating the eight focal countries, with τ ranging from March 31 to April 19, and using data up to April 20 (to compare the forecast and the actual data). Dotted curve: Proportions calculated when one only considers situations with at least 250 cumulative deaths at τ. Red curves are similar to curves displayed in Figure 2 in the main text.

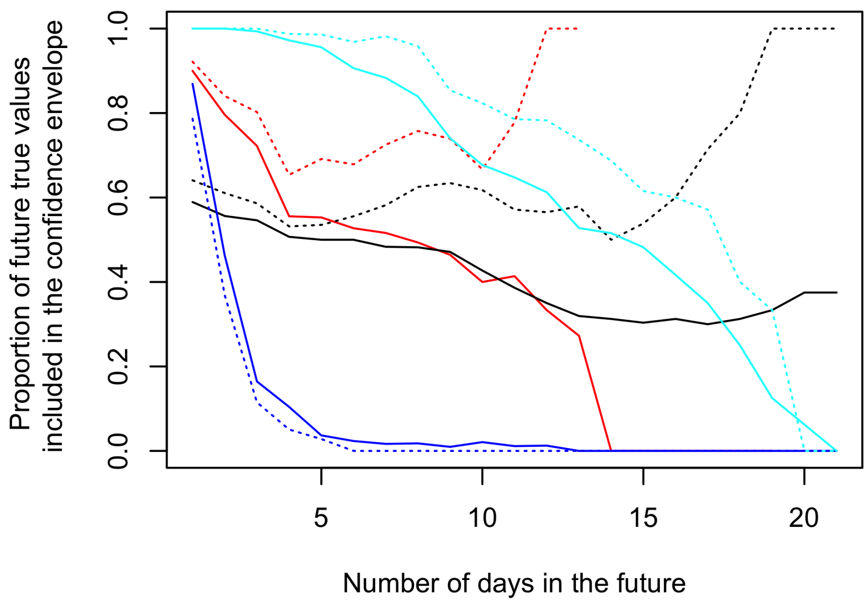

Supplement: S1 Data — (ZIP) [file pone.0238410.s001.zip › melange-Suppl_S4fig.pdf]
