# Supplementary material for: COVID-19 mortality dynamics: The future modelled as a (mixture of) past(s)
Source: PLoS One. 2020 Sep 11;15(9):e0238410. doi: 10.1371/journal.pone.0238410 (PMC7485826; doi:10.1371/journal.pone.0238410)

Figure S5. Estimated mixture probabilities across time for Austria and Sweden for a date of the last observation ranging from March 31 to April 20.

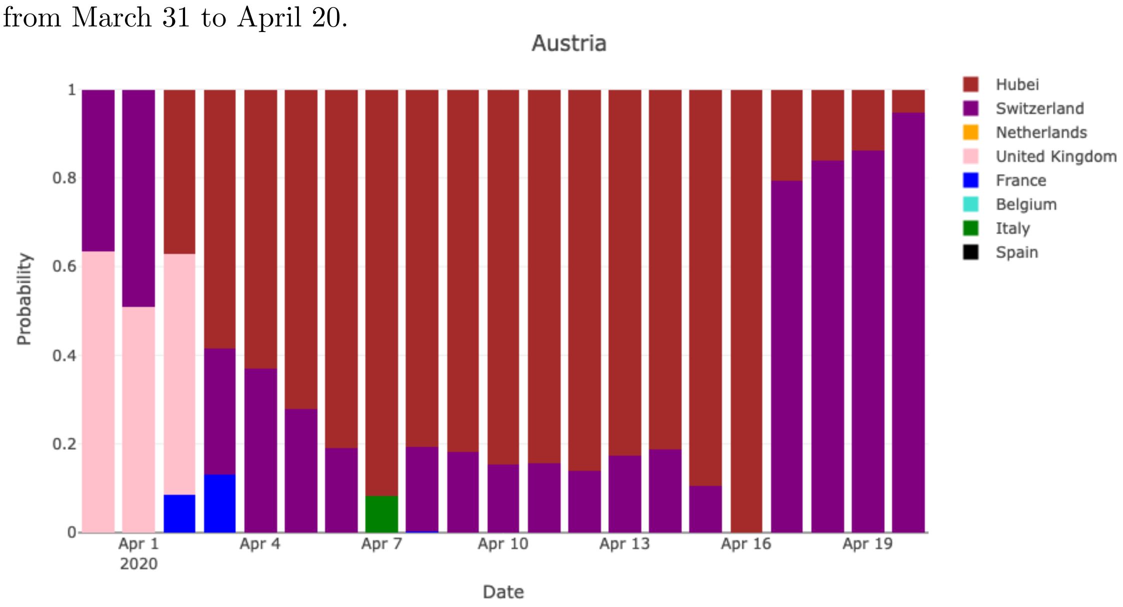

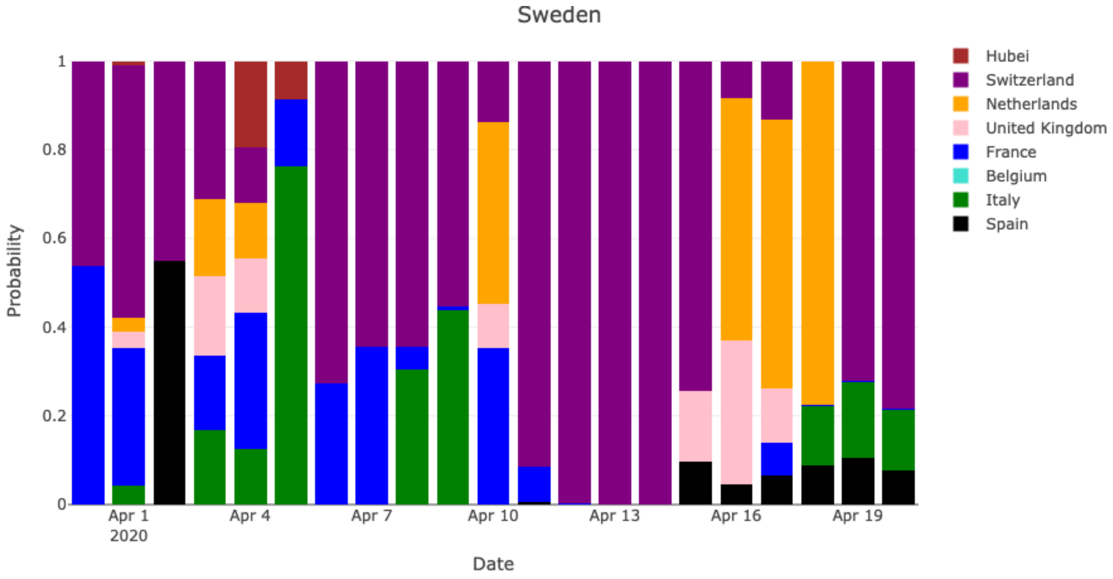

Supplement: S1 Data — (ZIP) [file pone.0238410.s001.zip › melange-Suppl_S5fig.pdf]
