# Supplementary material for: COVID-19 mortality dynamics: The future modelled as a (mixture of) past(s)
Source: PLoS One. 2020 Sep 11;15(9):e0238410. doi: 10.1371/journal.pone.0238410 (PMC7485826; doi:10.1371/journal.pone.0238410)

Figure S7. Mortality data scaled by population size (i.e. death rate) for 15 European countries and the Hubei province in China, up to April 20, 2020. The areas of disks in the legend are proportional to the population sizes. Data for front-line countries and second-line countries (as defined in the main text) are displayed in the top and bottom panels, respectively.

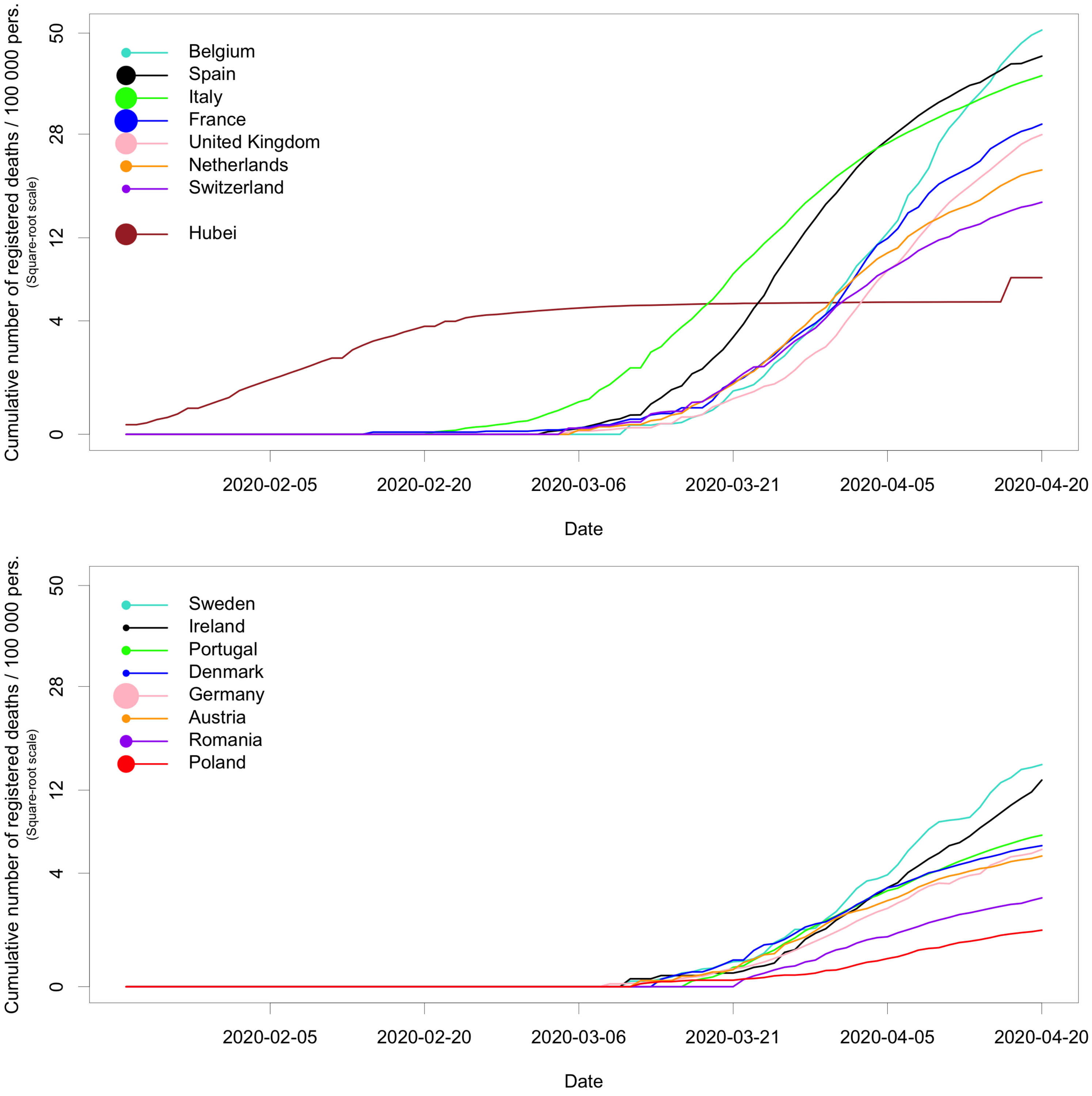

Supplement: S1 Data — (ZIP) [file pone.0238410.s001.zip › melange-Suppl_S7fig.pdf]
