# Supplementary material for: COVID-19 mortality dynamics: The future modelled as a (mixture of) past(s)
Source: PLoS One. 2020 Sep 11;15(9):e0238410. doi: 10.1371/journal.pone.0238410 (PMC7485826; doi:10.1371/journal.pone.0238410)

Figure S11. Forecast of the number of deaths from COVID-19 in Poland, as provided by the web app http://covid19-forecast.biosp.org/ on July 5, 2020. Top: Twenty predicting countries drawn in the set of countries in advance in terms of mortality rate were used as benchmark countries. Bottom: A parametric predictor (detailed in Supporting Text S1.4) fitted to Polish data is added to the 20 predicting countries in the mixture model.

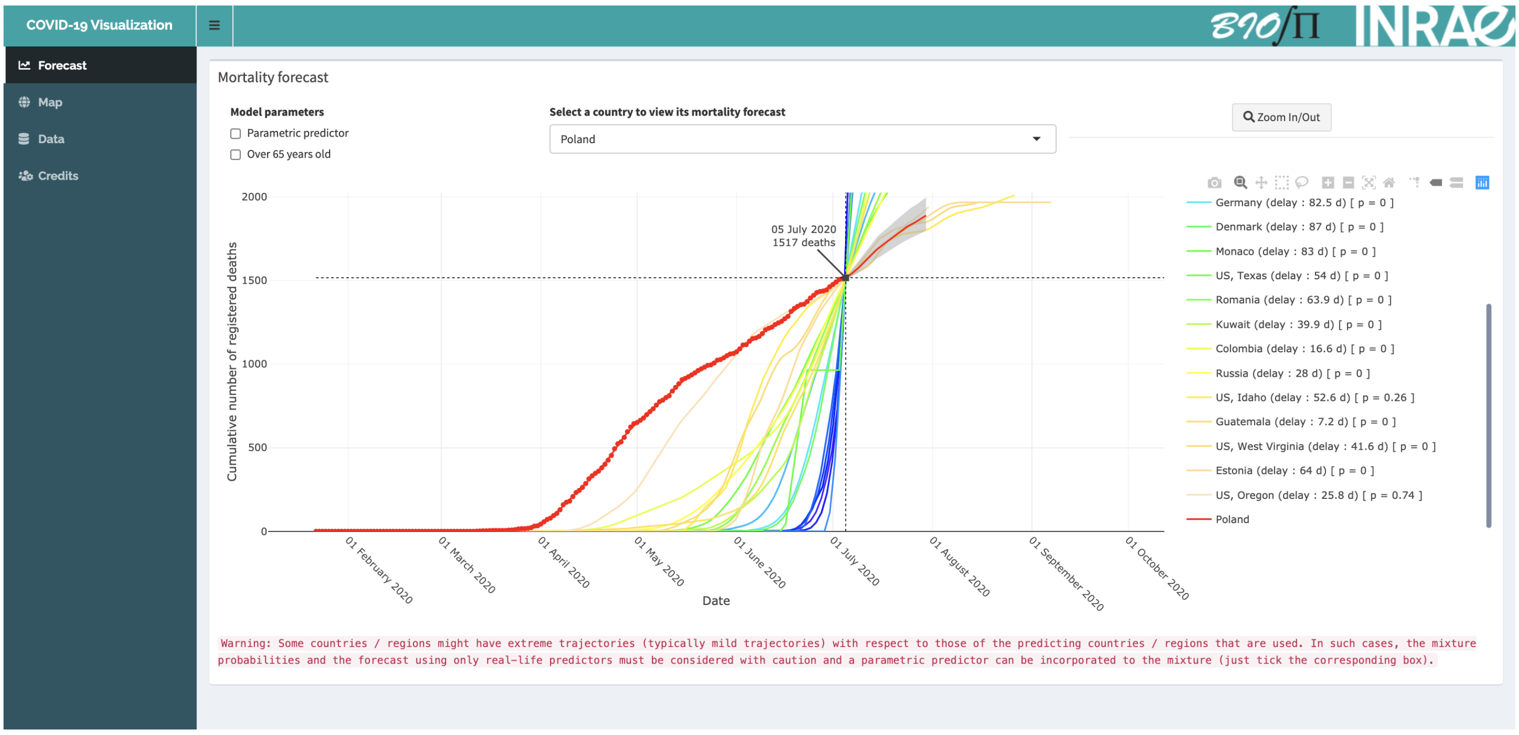

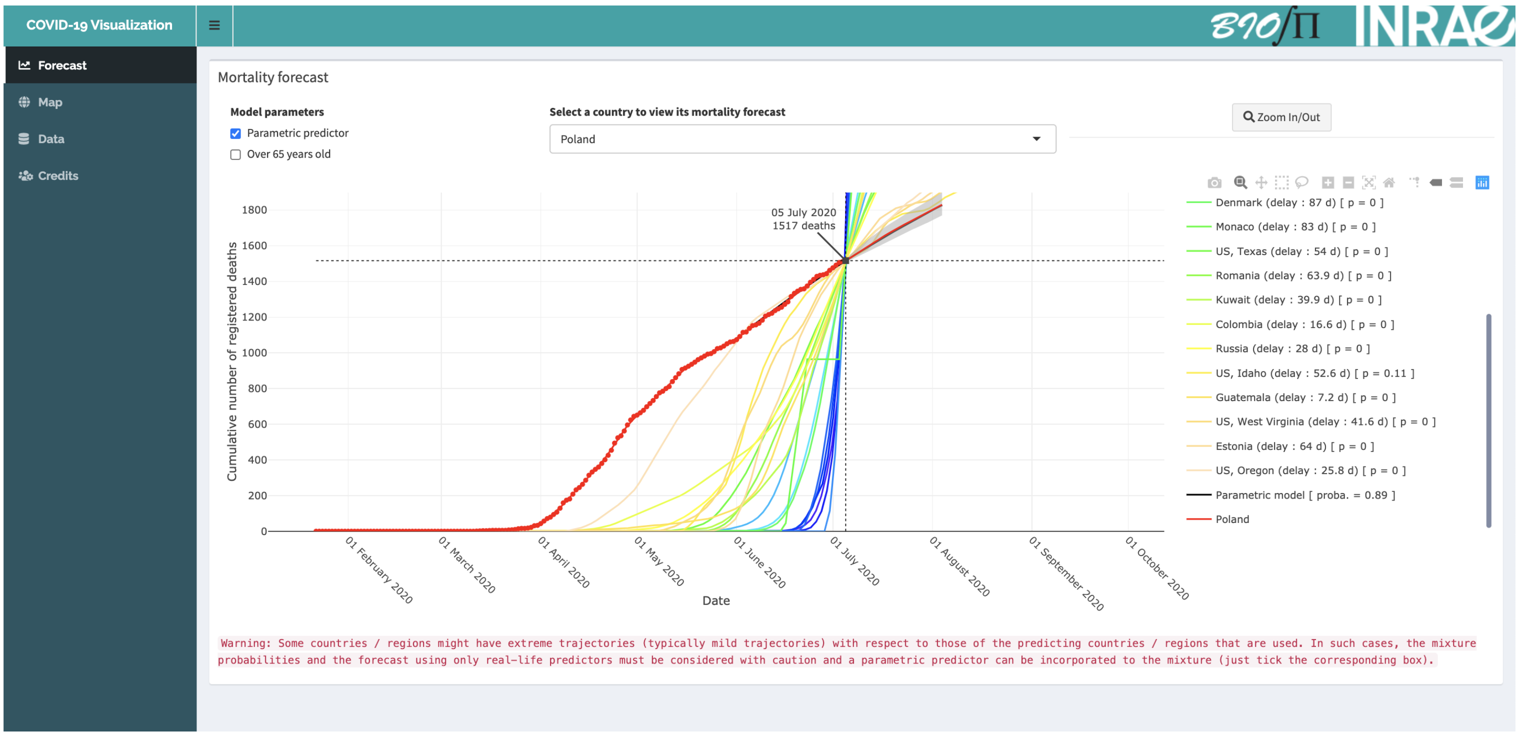

Supplement: S1 Data — (ZIP) [file pone.0238410.s001.zip › melange-Suppl_S11fig.pdf]
