# Supplementary material for: COVID-19 mortality dynamics: The future modelled as a (mixture of) past(s)
Source: PLoS One. 2020 Sep 11;15(9):e0238410. doi: 10.1371/journal.pone.0238410 (PMC7485826; doi:10.1371/journal.pone.0238410)

Figure S12. Forecast of the number of deaths from COVID-19 in Kyrgyzstan, as provided by the web app http://covid19-forecast.biosp.org/ on July 11, 2020, when the parametric predictor proposed in Supporting Texte S1.4 is added to the mixture.

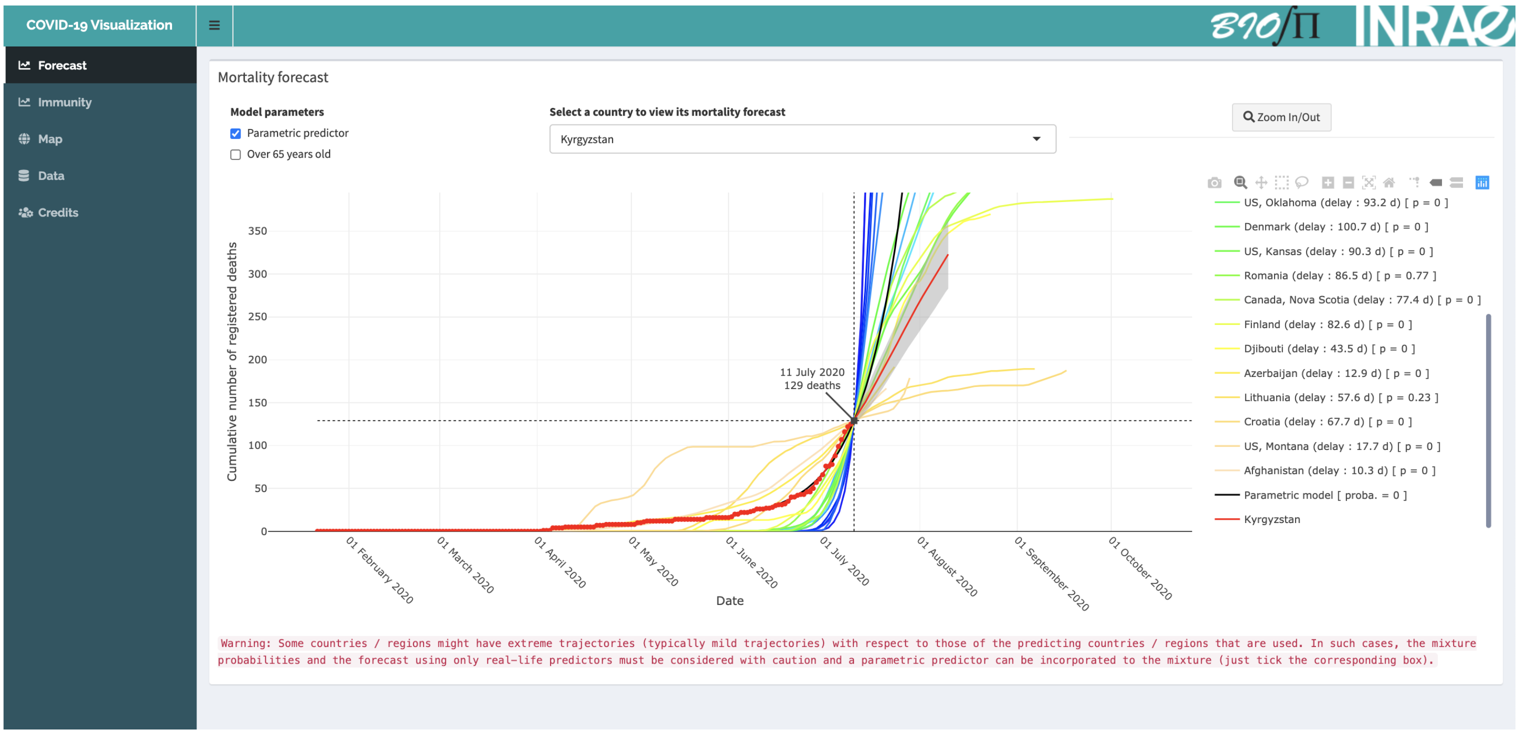

Supplement: S1 Data — (ZIP) [file pone.0238410.s001.zip › melange-Suppl_S12fig.pdf]
